# Adverse childhood experiences and mental health outcomes among women in sex work and social care: a cross-sectional study

**DOI:** 10.3389/fpubh.2026.1786872

**Published:** 2026-04-10

**Authors:** Franziska Kroehn-Liedtke, Anastasiia Lotysh, Olivia Kalinowski, Gizem Kaya, Hristiana Mihaylova, Martina Hernek, Wulf Rössler, Meryam Schouler-Ocak

**Affiliations:** Department of Psychiatry and Neurosciences, Psychiatric University Clinic of Charité at St. Hedwig Hospital, Berlin, Germany

**Keywords:** ACES, CTQ-SF, mental health, sex work, social care work

## Abstract

**Introduction:**

Adverse childhood experiences (ACEs) are well-established risk factors for mental disorders, yet their impact within vulnerable occupational groups remains insufficiently understood. Women engaged in sex work and social care are disproportionately exposed to violence and emotional stressors, potentially compounding the effects of early-life adversity.

**Methods:**

We conducted a mixed-methods study in Berlin, Germany, including 560 women (403 engaged in sex work, 157 in social care). Childhood adversity was assessed using the Childhood Trauma Questionnaire (CTQ-SF), and current psychiatric disorders were diagnosed via structured clinical interviews (MINI-DIPS). Severe ACE exposure was defined as moderate-to-severe trauma in at least two CTQ domains. Multivariable logistic regression models examined associations between ACE exposure and mental health outcomes, adjusting for age, socioeconomic indicators, and occupational stressors. In addition, 10 semi-structured interviews explored experiences with mental health care, coping strategies, and resilience.

**Results:**

Severe ACE exposure was reported by 47% of women in sex work and 16% of women in social care work. Higher ACE exposure was associated with increased odds of depression [adjusted OR (aOR) 3.8, 95% CI 2.3–6.3], panic disorder (aOR 2.7, 95% CI 1.6–4.7), post-traumatic stress disorder (aOR 5.7, 95% CI 3.1–10.7), and substance use disorders (aOR 4.0, 95% CI 2.3–7.1). Qualitative findings highlighted the interplay of early adversity and occupational stress, alongside resilience through peer support and advocacy.

**Discussion:**

ACEs substantially contribute to psychiatric morbidity among women in violence- and stress-exposed occupations. Trauma-informed approaches that integrate early-life adversity and occupational context are essential for prevention and care in these high-risk groups.

## Introduction

Adverse childhood experiences (ACEs) such as violence, abuse, or neglect are among the strongest predictors of poor mental health worldwide (1). Childhood trauma is recognized as a potential risk factor for psychiatric disorders in adulthood as post-traumatic stress disorders (PTSD), affective disorders, anxiety disorders, and substance use on a broad empirical basis (2). This evidence has prompted calls for early trauma prevention and trauma-informed health systems as central components of global mental health strategies (3). As a possible link between childhood trauma and the increased risk for mental illness, the increased vulnerability to reexperiencing violence among people with ACEs is discussed, leading to a significant contribution of ACEs to the global burden of disease, particularly among women, who are more frequently affected by violence (4, 5).

The association between ACEs and poor mental health is well documented in the general population, yet certain occupational groups that face disproportionately high risks remain understudied (6). Two of these groups are women engaged in sex work and those working in social care. Sex work refers to the provision of consensual sexual services in exchange for monetary compensation, including street, brothel, and online sex work, as well as self-employment. Social care encompasses paid professional activities in the social and community sectors, including social work, residential care, counseling, and support services for vulnerable populations.

Women continue to perform more emotionally intensive care work and are therefore more likely to experience moral injury, systematic coercion, and occupational gender dynamics (7, 8). Both groups face unique psychosocial stressors, structural inequalities, and high exposure to secondary trauma, yet are rarely studied in mainstream psychiatric research (9, 10). Women in sex work experience elevated rates of violence, stigma, and barriers to accessing health care, which are further compounded by a history of adversity (10, 11). Similarly, social care workers often face vicarious trauma and emotional distress, which may interact with their own adverse experiences (6).

Female sex workers (FSW) and women in social care are exposed to unique vulnerabilities. While both groups provide essential contributions to society through care provision or through often-stigmatized forms of labor, they are simultaneously at risk of structural violence, discrimination, and mental health stressors (12, 13). Evidence indicates that FSW report disproportionately high levels of depression, PTSD, substance use disorders (SUD) and suicidality compared with the general population (14, 15). However, few studies have examined how much of this burden can be attributed to earlier childhood adversity vs. occupational stress. In social care, research has largely focused on occupational stress and burnout, but not on the cumulative effect of ACEs in shaping vulnerability to poor mental health (6). Moreover, most existing studies are small, fragmented, and regionally limited, with little integration of quantitative and qualitative perspectives (10, 12). Importantly, lived experiences and voices of women affected are rarely incorporated into psychiatric research, despite calls from patient advocacy groups and international policy frameworks for greater participation and co-production (16).

This lack of integrated evidence has major implications. Without understanding how ACEs intersect with occupational exposures in sex work and social care, clinicians, policymakers, and service providers cannot design effective prevention and intervention strategies. Ignoring the specific needs of these populations risks perpetuating health inequities and undermining progress toward trauma-informed systems of care. In the context of rising global concern about mental health among vulnerable populations, there is an urgent need to close this evidence gap.

In this study, we aimed to examine the association between ACEs and mental health outcomes among women engaged in sex work and social care. Using a mixed-methods approach, we combined validated quantitative measures of ACEs, and mental disorders like SUD, depression, anxiety, and PTSD with qualitative interviews that captured lived experiences of barriers in mental health services and resilience. By triangulating findings, we sought to provide a more nuanced understanding of how early adversity and occupational stress interact to shape mental health trajectories. Our objective was not only to inform psychiatric care, but also to generate evidence relevant to social policy and public health, with a focus on reducing stigma and improving service access for these marginalized groups.

## Methods

### Study design and participants

We conducted a cross-sectional study in Berlin, Germany to examine the association between adverse childhood experiences (ACEs) and mental health outcomes among women engaged in sex work and social care. This study employed an explanatory sequential mixed-methods design combining quantitative survey data with qualitative semi-structured interviews. The quantitative component examined associations between ACEs and mental health outcomes, while the qualitative interviews aimed to provide contextual insight into the lived experiences of the participant sex workers. Interviews were analyzed using thematic analysis, with transcripts coded inductively and themes refined through iterative discussions within the research team. Integration of quantitative and qualitative findings occurred during the interpretation phase, where qualitative results were used to contextualize and enrich the interpretation of the quantitative findings.

Between June, 2022 and November, 2024, we recruited participants from community organizations, professional networks, and outreach services. Eligible participants were women aged 18 years or older who were engaged in either sex work or social care occupations. Therefore, participants from various areas of sex work were included, such as street, brothel, and online sex as well as escort services, stripping, webcam modeling and pornography. The term “social care workers” refers to professionally trained social workers and related care professionals employed in social and community-based services. In this study, the social care workers were almost exclusively women with a university degree in social work or social pedagogy who worked in various counseling centers, health facilities, and social projects, such as homelessness services or street outreach. We excluded individuals unable to provide informed consent because of severe cognitive impairment, psychotic decompensation, and acute suicidality or those with insufficient language proficiency to complete assessments.

A non-proportional quota sampling approach was used to recruit women engaged in sex work and social care. Quotas were defined a priori to ensure adequate representation of both occupational groups. As a non-probability sampling strategy, this approach does not permit population-level inference but was chosen to facilitate access to hard-to-reach populations and enable comparisons between occupational groups. To maximize participation, sex workers were recruited through direct outreach at multiple work settings, including street-based locations, studios, brothels, and escort services, as well as via social media platforms (e.g., Instagram). Study information was distributed at these sites to facilitate engagement. Recruitment was additionally supported by specialized counseling and advisory centers for sex workers, as well as organizations providing services to individuals experiencing homelessness. The comparison group of social care professionals was recruited online through counseling and professional networks across Germany and via personal contacts. Recruitment in both groups was primarily purposive and supported by informal snowballing, as participants occasionally shared study information with colleagues or peers.

No formal a priori power calculation for all study outcomes was conducted. Initial feasibility considerations were informed by expected differences in ACE prevalence between occupational groups based on prior literature. To allow multivariable analyses with adjustment for key covariates, subgroup analyses, and potential clustering by recruitment site, the target sample size was substantially increased. Allowing for incomplete or invalid responses, a final sample size of approximately 500 participants was considered sufficient to provide stable estimates for the primary analyses. Recruitment was conducted over a predefined study period rather than until a strict numerical threshold was reached. All eligible participants who consented during this period were included in the final sample, resulting in a total of 560 participants. The larger sample size improved statistical precision but did not alter the predefined analytic strategy.

### Procedures

After providing informed consent, participants completed structured questionnaires administered by trained female research assistants in private settings in face-to-face meetings or online. Female research assistants were selected because the study addressed sensitive topics such as trauma, violence, and sex work, and the use of same-gender interviewers was intended to enhance participant comfort and facilitate open disclosure. Data collection captured sociodemographic characteristics (age, nationality, residence status, education, income), occupational factors (years in current role, location and setting of work, exposure to work-related violence), and childhood adversity.

Psychiatric diagnoses were assessed with the MINI-DIPS, a structured interview aligned with DSM-IV and ICD-10 criteria (17). The instrument covers major psychiatric disorders, including mood, anxiety, substance use, and somatoform disorders, and has demonstrated high interrater reliability (κ > 0.80 for most diagnoses) and good convergent validity with other structured interviews (e.g., SCID). ACEs were assessed using the Childhood Trauma Questionnaire (CTQ-SF), which covers five domains (emotional, physical, and sexual abuse; emotional and physical neglect) (18). Items are rated on a 5-point Likert scale (1 = never true to 5 = very often true). The CTQ-SF includes a minimization/denial scale to detect underreporting and shows excellent reliability (α > 0.90 total; α > 0.70 subscales) and good construct validity. Higher scores reflected greater symptom severity, with established cutoffs indicating probable cases.

To ensure reliability, all instruments had been previously translated in the study population's language(s) including German, English, Russian, Bulgarian, Ukrainian, Polish, Romanian, Turkish, Vietnamese and Hungarian. Translations followed a forward–backward translation procedure. Instruments were translated by bilingual native speakers of the research staff and subsequently back-translated by independent native speakers not involved in the study. Discrepancies were reviewed and resolved by the research team to ensure semantic equivalence. Interviews were conducted in the native language of the participants.

### Outcomes

#### ACE exposure

Childhood adversity was assessed using the Childhood Trauma Questionnaire (CTQ-SF), which comprises five subscales (emotional, physical, and sexual abuse; emotional and physical neglect). Subscale scores were categorized into four severity levels (none, low, moderate, severe) according to Bernstein and Fink's established thresholds (19). Severe ACE exposure was defined as moderate-to-severe scores in at least two CTQ subscales. In addition, the CTQ total score (range 25–125) was analyzed as a continuous measure of cumulative adversity.

#### Mental health outcomes

Current and lifetime psychiatric diagnoses were assessed using the MINI-DIPS structured clinical interview, covering diagnostic categories according to DSM-IV and ICD-10 criteria (17). Binary outcome variables were created for the presence of any current psychiatric diagnosis, as well as for specific diagnostic categories (depressive disorders, panic disorder, PTSD, substance use disorders).

#### Sensitivity analyses

As an alternative operationalization, ACE exposure was categorized based on the number of CTQ subscales with at least moderate severity (0–1, 2–3, ≥4 out of five domains), allowing comparison with commonly used ACE count approaches. ACEs were categorized into three groups to reflect increasing cumulative exposure and to allow for examination of potential dose–response patterns. This categorization preserves more information for distinguishing between low, moderate, and high adversity exposure than a simple dichotomization (e.g., 0 vs. ≥4) as used in some large epidemiological studies, which can obscure intermediate risk gradients and reduce statistical power.

All outcomes were additionally examined separately for women engaged in sex work and those employed in social care to allow exploratory group comparisons. Missing data were handled using listwise deletion.

### Qualitative component

A targeted sample of 10 participants was invited to conduct semi-structured qualitative interviews to investigate the relationship between work-related stressors, barriers to healthcare, and coping strategies. The interviews were conducted in person or online and lasted 60–90 min. Interviewers followed a guideline that included life experiences, perceived links between stressors and current mental health, and resilience and support strategies. All qualitative interviews were recorded with the participants' consent, transcribed verbatim, and anonymized. The transcripts were checked for accuracy and translated into English where necessary. The interview sample size was guided by the exploratory nature of the qualitative component and aimed to capture individual perspectives in the group of FSW. Data collection continued until thematic saturation was reached, defined as the point at which no substantially new themes emerged from additional interviews. After the final interviews, the research team determined that the main thematic patterns had been sufficiently captured and therefore closed data collection.

### Statistical analysis

We summarized participant characteristics using means (SD) for continuous variables and frequencies (%) for categorical variables. Differences between sex work and social care groups were assessed using χ^2^ tests or *t* tests, and Mann-Whitney *U*-test for non-normally distributed data, as appropriate.

Associations between ACE exposure and mental health outcomes were analyzed using multivariable logistic regression models. Primary models used the CTQ-based definition of severe ACE exposure. Sensitivity analyses applied alternative ACE categorizations based on the number of affected domains (0–1, 2–3, ≥4).

All models were adjusted for age, education, income, and exposure to occupational physical violence. Adjusted odds ratios (aORs) with 95% confidence intervals (CIs) were reported. Wide confidence intervals were interpreted as indicating limited precision.

A two-sided *p* value < 0.05 was considered statistically significant. All analyses were conducted in SPSS, Version 31.0.0.0.

### Qualitative analysis

Interview transcripts were analyzed in MAXQDA 2022 using structuring qualitative content analysis following Kuckartz. A combined deductive–inductive approach was applied. Deductive main categories were derived from the trauma-informed care (TIC) model (20) and the interview guide, while inductive subcategories emerged from the data (e.g., barriers in TIC, stigma experiences, coping strategies).

Coding proceeded iteratively: after an initial round of open coding, the category system was refined and applied to all transcripts.

To ensure transparency and reliability, a detailed codebook defined all categories and inclusion criteria. Two researchers independently coded a subset of transcripts; discrepancies were discussed and resolved through consensus meetings, resulting in a refined final coding framework. The qualitative interviews were part of a larger qualitative study previously reported (21).

Triangulation with quantitative findings was used to contextualize associations and highlight mechanisms linking childhood adversity to current mental health.

### Ethics approval and consent

The study was approved by the ethics commission of Charité – Universitätsmedizin Berlin (EA2/133/18). All participants provided written informed consent before participation. Given the vulnerability of the study population, confidentiality safeguards were prioritized: identifiers were stored separately from data, interview transcripts were anonymized, and participants were offered referrals to psychological support services if required.

## Results

### Participant characteristics

A total of 560 women participated in the study, including 403 women engaged in sex work and 157 in social care. Data from two participants had to be excluded due to withdrawal of consent. The mean age was 33.8 years (*SD 9.2*) among the sex workers and 35.5 years (*SD 10.2*) for the social care workers. Most participants identified as cisgender women (92.5% in the sex worker sample and 96.8% of the social care workers), and 41.2% of the FSW reported a migration background whereas only 4.5% of the social care workers. Women in sex work had, on average, lower formal education levels (62.4% completed secondary education and 22.8% had a university degree vs. 89.8% in social care) and higher rates of financial insecurity (monthly income > 1,000 €: 60.9% among the FSW vs. 94.8% in social care).

Exposure to workplace violence in the last six months was more frequently reported by the FSW: 12.3% experienced physical violence, 10.4% sexual violence, whereas only physical violence was reported by 9.9% of the participants in social care. Table 1 shows the sociodemographic and clinical profile of the two study groups.

**Table 1:** Sociodemographic and clinical characteristics of the sample (*N* = 560).

Characteristic	Sex work (*n* = 403)	Social care work (*n* = 157)	*p*-value
Age, mean (SD)	33.8 (SD = 9.2)	35.5 (SD = 10.2)	0.117
Migration background, *n* (%)	168 (41.2 %)	7 (4.5 %)	0.005
Secondary education, *n* (%)	251 (62.4 %)	141 (89.8 %)	< 0.001
Monthly income > 1,000, *n* (%)	241 (60.9 %)	149 (94.8 %)	< 0.001
CTQ total, mean (SD)	49.3 (SD = 19.8)	39.1 (SD = 12.6)	< 0.001
Depression, *n* (%)	84 (20.9 %)	15 (9.6 %)	0.018
Anxiety disorder, *n* (%)	72 (18.0 %)	12 (7.7 %)	0.005
PTSD, *n* (%)	68 (17.0 %)	3 (1.9 %)	< 0.001
SUD, *n* (%)	79 (19.7 %)	1 (0.6 %)	< 0.001
Physical violence in adulthood, *n* (%)	49 (12.3 %)	15 (9.6 %)	0.430

Values are mean (SD) or n (%). *p* values derived from Mann-Whitney *U*-test (for age) or χ^2^ tests as appropriate. Table 1 shows the sociodemographic and clinical profile of the two study groups. Women in sex work reported higher childhood trauma and greater prevalence of psychiatric diagnoses compared with women in social care work.

### Childhood trauma exposure

High levels of childhood trauma were common in both groups. Mean total CTQ scores were significantly higher among women in sex work [mean 49.3 (SD 19.8)] than among those in social care [39.1 (12.6); *t*_(437)_ = 7.12, *p* < 0.001], representing a medium effect size (Cohen's *d* = 0.61). Severe exposure (≥ moderate in ≥ 2 subscales) was observed in 47.4% of the sex work group and 16.8% of the social care group.

Among FSW emotional abuse (39.1%) was the most frequent trauma type (≥ moderate), followed by emotional neglect (36.4%), physical neglect (34.4%), sexual abuse (33.8%), and physical abuse (23.5%). In the group of social care workers sexual abuse (24.5%) was the dominating trauma type, followed by emotional abuse (20.0%), emotional neglect (12.9%), physical neglect (12.3%), and physical abuse (5.2%).

### Psychiatric diagnoses

According to MINI-DIPS, 60.3% of the FSW and 44.2% of the participants in social care met diagnostic criteria for at least one current mental disorder (*p* = 0.001).

Major depressive disorder was present in 20.9% of sex workers compared to 9.6% of social care workers (*p* = 0.018). Panic disorder was diagnosed in 18.0% vs. 7.7% (*p* = 0.005), post-traumatic stress disorder in 17.0% vs. 1.9% (*p* < 0.001), and substance use disorders in 19.7% vs. 0.6% (*p* < 0.001).

Comorbidity was common: In the sex worker sample 39.9% of those with a diagnosis met criteria for two or more disorders whereas 18.6% in the social care group (*p* < 0.001).

### Associations between ACEs and mental disorders

Higher CTQ total scores were strongly associated with the presence of a current psychiatric diagnosis. In adjusted logistic regression models controlling for group, age, education, income, and workplace physical violence, each 10-point increase in CTQ score was associated with a 63.7% increase in the odds of having a diagnosis (aOR = 1.64; 95% CI 1.02–2.64; *p* = 0.043).

High trauma exposure, defined as CTQ scores in the moderate-to-severe range in at least two domains, was significantly associated with higher odds of all examined diagnoses, including depression (aOR = 3.87; 95% CI 2.36–6.33; *p* < 0.001), panic disorder (aOR = 2.78; 95% CI 1.63–4.75; *p* < 0.001), post-traumatic stress disorder (aOR = 5.79; 95% CI 3.13–10.71; *p* < 0.001), and substance use disorder (aOR = 4.06; 95% CI 2.31–7.12; *p* < 0.001).

Stratified logistic regression analyses were conducted separately for FSW and social care workers. For sex workers, a higher score for childhood trauma was associated with an increased likelihood of a current psychiatric diagnosis (aOR = 1.75, 95% CI: 0.96–3.17), although this association was not statistically significant. No other predictors showed a significant association with the outcome. Estimates for social care workers were unstable due to small cell sizes in some categories.

The interaction between CTQ severity and occupational group was not statistically significant (aOR = 0.84; 95% CI 0.62–1.12; *p* = 0.229), although the direction of the effect suggested a comparatively weaker association between adverse childhood experiences and mental disorders among women in social care compared to female sex workers.

**Figure 1:**
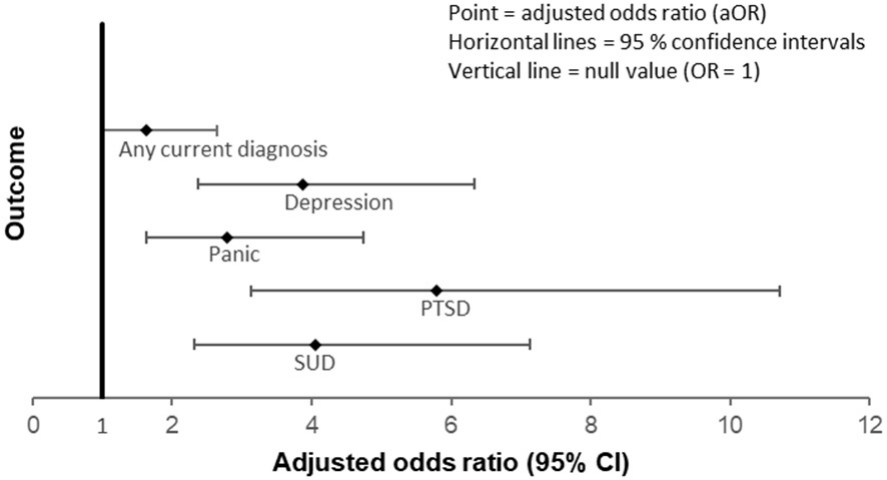
Adjusted odds ratios (aORs) for psychiatric disorders by occupational group and trauma exposure. Forest plot showing adjusted odds ratios (aORs, 95% CIs) for any psychiatric disorder, depression (MD), panic disorder, post-traumatic stress disorder (PTSD), and substance use disorder (SUD) associated with childhood trauma severity (Childhood Trauma Questionnaire total score, per 10-point increase) and high trauma exposure (≥ moderate in ≥ 2 domains), stratified by occupational group (sex work vs. social care). Models were adjusted for age, education, and income. The vertical line represents the null value (aOR = 1). Error bars indicate 95% confidence intervals.

In unadjusted analyses, women engaged in sex work showed higher odds of current psychiatric disorders compared with women in social care; this association attenuated after multivariable adjustment and was accompanied by wide confidence intervals. Table 2 summarizes associations between cumulative childhood trauma and psychiatric morbidity. Table 3 presents the interaction model. Figure 1 displays adjusted odds ratios (aORs, 95% CIs) for the main psychiatric outcomes.

**Table 2:** Association between childhood trauma (CTQ total score) and presence of any psychiatric diagnosis.

Variable	aOR (95% CI)	*p*-value	FSW aOR (95% CI)	*p*-value	Social care worker aOR (95% CI)	*p*-value
CTQ total (per 10 points)	1.64 (1.02–2.64)	0.043	1.75 (0.96–3.17)	0.068	1.24 (0.41–3.73)	0.706
Age (years)	1.02 (0.95–1.10)	0.587	1.00 (0.90–1.10)	0.963	1.25 (0.94–1.66)	0.126
Education (ref = high)	0.62 (0.08–4.61)	0.637	0.56 (0.07–4.72)	0.595	–	–
Income (ref = stable)	1.64 (0.61–4.39)	0.325	1.92 (0.59–6.24)	0.277	0.02 (0.00–4.37)	0.152
Physical violence in adulthood (yes vs. no)	1.02 (0.77–1.36)	0.871	1.07 (0.79–1.44)	0.680	–	–

Adjusted odds ratios (aOR) from multivariable logistic regression controlling for listed covariates. Table 2 summarizes the association between cumulative childhood trauma and psychiatric morbidity for the total sample and in the two groups. For FSW higher CTQ scores were associated with increased odds of any mental disorder after adjustment for sociodemographic and violence-related factors.

**Table 3:** Interaction between childhood trauma and occupational context on mental health outcome.

Predictor	aOR (95 % CI)	*p*-value
CTQ total (centered)	1.78 (1.23–2.59)	0.002
Group (sex vs. social care work)	1.66 (0.48–5.77)	0.423
CTQ × Group interaction	0.84 (0.62–1.12)	0.229

Logistic regression including centered CTQ, group, and their interaction term. Table 3 presents the interaction model, suggesting that the effect of childhood trauma on psychiatric morbidity may be more pronounced among women in sex work.

### Qualitative findings

From the qualitative interviews, four overarching themes emerged.

#### Cycles of trauma and vulnerability

Many participants described recurrent exposure to violence and deprivation, both in childhood and adulthood. Several interviewees reflected on how early experiences shaped later vulnerability.

“*It's difficult to go to a new place and like childhood traumas and explain to them why I'm there. [...] I like have to feel like I have traumas that I have to do with this, this and this, and that's incredibly difficult. And I have to do it again and again and again. It's that, yeah. So I find it difficult.” (FSW, Age 35, Icelandic)*

#### Stigma and isolation

Early trauma and occupational stigma compounded feelings of self-stigmatization and isolation.

“*People judge you. And it was actually more of an issue with me being a sex worker. It's quite embarrassing.” (FSW, Age 29, Italian)*

“*I'm fearful for it that there's judgments or that, they don't give the same level of care in a way that it would affect, whether they realize it or not as well. I think sometimes it can be subconscious bias also.” (FSW, Age 34, English)*

#### Resilience through peer networks

Informal support among colleagues and community networks provided crucial emotional stability. Many participants emphasized the importance of informal peer support networks that helped them cope with difficult experiences.

“*…we have a good team we talk to each other in our studio with my colleagues […] sometimes you want to talk about clients or about this stuff with someone who will understand you even.” (FSW, Age 25, Russian)*

#### Gaps in trauma-informed care

Participants reported negative experiences with health services lacking sensitivity to trauma histories.

“*…a psychiatrist isn't projecting or assuming stereotypes of what they have, which can be a barrier to what I'm actually saying or for, to them to hear what I'm saying […] I think like the most important thing for accessing care, I guess, is that like, okay, you can hear what I'm saying, you‘re listening to me and you don't think, you know, better than what I'm saying, or you're not trying to find out something that I'm not telling you.” (FSW, Age 29, Australian)*

**Figure 2:**
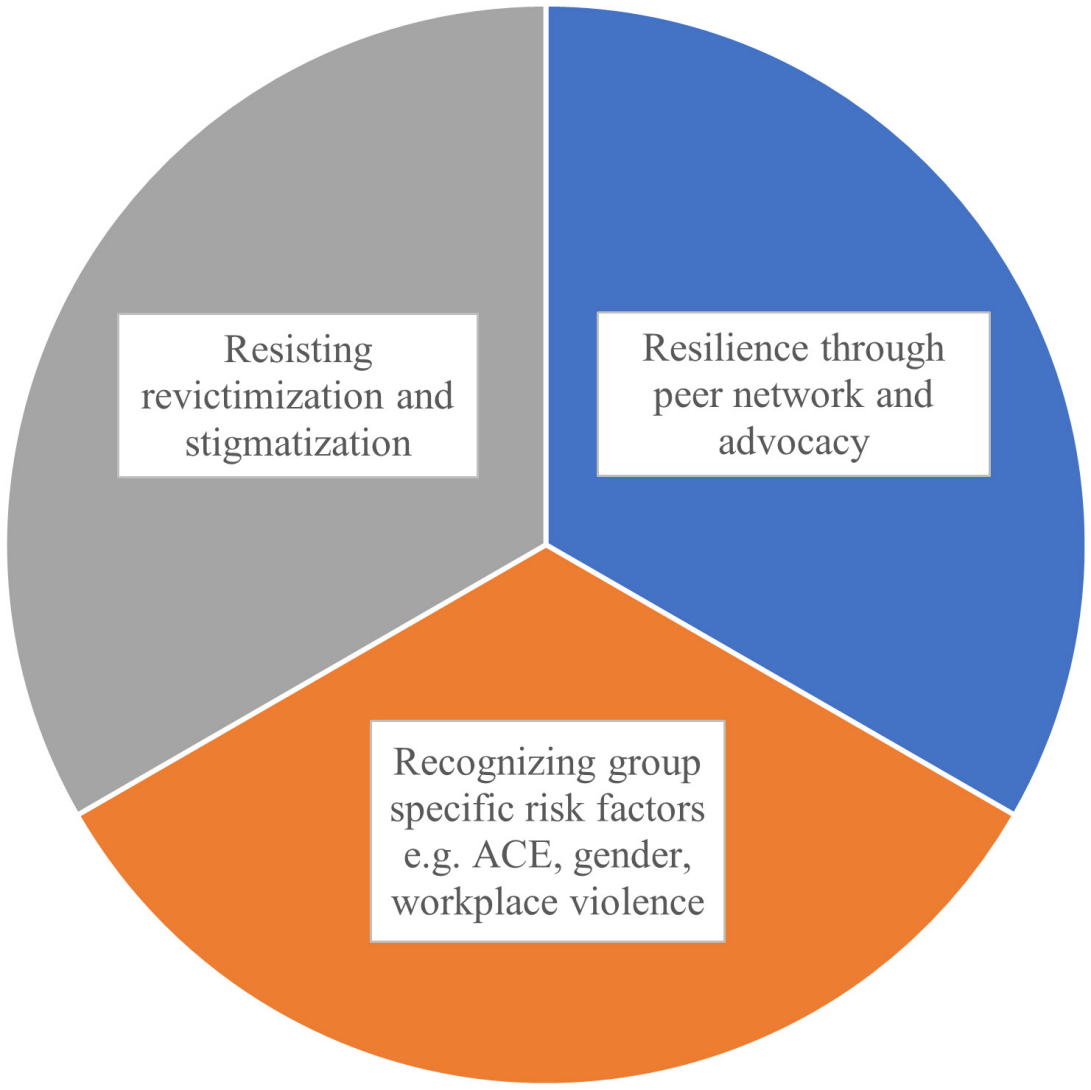
Conceptual themes derived from qualitative analysis within a trauma-informed framework. The figure illustrates three interrelated domains identified in the qualitative analysis that reflect trauma-informed principles as outlined by the Substance Abuse and Mental Health Services Administration [SAMHSA, (34)]. Key aspects include (1) recognizing group-specific risk factors such as adverse childhood experiences (ACEs), gender-based and workplace violence, highlighting the need for awareness of structural determinants and safety; (2) resisting revictimization and stigmatization, which reflects empowerment and the creation of trust-based environments that reduce retraumatization; and (3) resilience through peer networks and advocacy, aligning with SAMHSA's principles of peer support, collaboration, and empowerment. Together, these domains demonstrate how trauma-informed approaches can enhance psychosocial wellbeing and foster recovery among women exposed to intersecting vulnerabilities.

To synthesize these findings, Figure 2 presents the interrelated domains emerging from the qualitative analysis and highlights their implications for trauma-informed care.

Integration of quantitative and qualitative data indicated that early trauma not only increased psychiatric risk directly but also shaped life trajectories marked by revictimization and reduced access to supportive systems.

### Key summary

High rates of childhood trauma and psychiatric morbidity were observed among women in both occupational groups, particularly those engaged in sex work. ACEs were strongly linked to mental disorders and partially mediated by occupational stressors such as violence. The qualitative findings highlighted the cyclical nature of trauma exposure and the protective role of peer support.

## Discussion

In this mixed-methods study of women engaged in sex work and social care, adverse childhood experiences (ACEs) were highly prevalent and strongly associated with current psychiatric disorders. Women engaged in sex work reported particularly high levels of childhood trauma and a substantial burden of mental disorders, especially depression, post-traumatic stress disorder (PTSD), and substance use disorders (SUD). The qualitative findings helped contextualize the quantitative associations between childhood trauma and mental health outcomes. Overall, the findings extend existing evidence linking early adversity to adult psychopathology and underscore the importance of considering occupational context when examining mental health outcomes in vulnerable populations.

### Summary of main findings

Approximately two thirds of women engaged in sex work and nearly half of those employed in social care met criteria for at least one current mental disorder. Severe childhood trauma exposure, defined using established CTQ thresholds, was substantially more common among women in sex work than among social care workers. Across both occupational groups, higher CTQ scores were consistently associated with increased odds of depression, panic disorder, PTSD, and SUD, supporting a dose–response relationship between cumulative childhood adversity and psychiatric morbidity.

Although women in sex work experienced higher levels of both childhood trauma and workplace violence, the interaction between ACE severity and occupational group was not statistically significant. This suggests that the association between childhood adversity and adult mental disorders was broadly comparable across groups, albeit occurring in different occupational risk environments. Some regression estimates —particularly for occupational group in fully adjusted models— were accompanied by wide confidence intervals, indicating limited precision and the potential influence of sparse data.

Qualitative findings provided important contextual insight into these quantitative associations. Participants described overlapping trajectories of early abuse, adult victimization, and barriers to accessing mental health care, including stigma and mistrust of services. At the same time, many women articulated sources of resilience, such as peer support, community networks, and engagement in advocacy, highlighting heterogeneity in responses to adversity. Taken together, the quantitative and qualitative findings support the interconnected domains illustrated in Figure 2, particularly the specific risk factors as traumatic childhood adversity, the importance of peer support and the need to prevent revictimization in trauma-informed care.

### Comparison with previous research

The present findings are consistent with a substantial body of international research demonstrating that ACEs contribute markedly to the global burden of mental disorders, particularly among women and socially marginalized groups (22, 23). Large population-based studies have shown that exposure to multiple forms of childhood adversity increases the lifetime risk of affective, anxiety, and substance use disorders by 2- to 4-fold, often in a graded manner (24–26). Evidence also indicates that early trauma enhances vulnerability to revictimization and interpersonal violence in adulthood (27, 28).

Women engaged in sex work have been identified as a particularly vulnerable population worldwide, with high exposure to violence, stigma, and structural discrimination (29, 30). The elevated prevalence of both childhood and adult violence among women in sex work echoes findings from global meta-analyses showing extreme rates of trauma exposure in this population (10, 11, 31, 32). Less severe but qualitatively similar patterns have been reported among care professionals, whose work is characterized by sustained emotional labor, secondary trauma, and elevated risk of burnout (33). The convergence of early adversity with occupational stress may therefore represent a cumulative pathway to poor mental health that remains insufficiently addressed in prevention and policy frameworks.

### Strengths and limitations

This study benefits from a large sample, the use of validated diagnostic instruments (CTQ, MINI-DIPS), and a mixed-methods design that combines quantitative associations with qualitative contextualization. The comparison of two female-dominated but socio-occupationally distinct groups provides insight into how childhood trauma and occupational environments jointly shape mental health outcomes.

Several limitations should be considered. The cross-sectional design precludes causal inference. Childhood trauma was assessed retrospectively and may be affected by recall bias, although the CTQ minimization scale mitigates this concern. The use of non-probability sampling through community organizations may limit generalizability, particularly to women not connected to services. Finally, despite multivariable adjustment, residual confounding by unmeasured factors (e.g., genetic vulnerability) cannot be excluded.

### Implications for practice and policy

The findings underscore the need for trauma-informed mental health care and occupational health systems, particularly for women in high-stress or violence-exposed professions. Trauma-informed frameworks emphasize safety, empowerment, and recognition of trauma histories across service settings —principles increasingly endorsed in WHO and UN guidance on global mental health transformation (34–36). Early identification of ACEs and the integration of trauma-informed principles into adult mental health care may help reduce chronic psychiatric morbidity and interrupt cycles of revictimization (22).

At a policy level, the high prevalence of trauma across both occupational groups underscores the importance of coordinated prevention strategies spanning childhood protection, workplace safety, and gender-based violence reduction. Embedding trauma-informed training within health and social care professions may also enhance workforce resilience and retention.

### Future research

Longitudinal studies are needed to clarify causal pathways linking childhood adversity, occupational stress, and adult mental health outcomes. Future research should also examine protective and resilience-promoting factors—such as peer support, self-advocacy, and access to responsive services—that may buffer the effects of high ACE exposure. Mixed-methods designs will remain essential for capturing the complex interplay between individual experience and structural determinants of mental health.

## Conclusion

This study contributes to growing evidence that childhood adversity plays a substantial role in shaping adult mental health, particularly among women exposed to occupational stress and violence. The findings highlight how early-life trauma intersects with occupational stress and violence to shape mental health outcomes in vulnerable female-dominated professions. Addressing these intersecting vulnerabilities is critical for reducing psychiatric morbidity and promoting mental wellbeing in high-risk occupational groups.

## Data Availability

The datasets presented in this article are not readily available because of the sensitive nature of the data. They can be accessed upon request from the authors and with appropriate ethical approval. Requests to access the datasets should be directed to meryam.schouler-ocak@charite.de.
